# Effects of Repeated Sublethal External Exposure to Deep Water Horizon Oil on the Avian Metabolome

**DOI:** 10.1038/s41598-018-36688-3

**Published:** 2019-01-23

**Authors:** Brian S. Dorr, Katie C. Hanson-Dorr, Fariba M. Assadi-Porter, Ebru Selin Selen, Katherine A. Healy, Katherine E. Horak

**Affiliations:** 1 US Department of Agriculture, Wildlife Services, National Wildlife Research Center, MS State, MS 39762 USA; 2 Department of Integrative Biology, University of Wisconsin-Madison, Madison, Wisconsin 53706 USA; 3 US Fish and Wildlife Service, Deepwater Horizon Natural Resource Damage Assessment and Restoration Office, Fairhope, AL 36532 USA; 4 US Department of Agriculture, Wildlife Services, National Wildlife Research Center, Fort Collins, CO 80521 USA

## Abstract

We assessed adverse effects of external sublethal exposure of Deepwater Horizon, Mississippi Canyon 252 oil on plasma and liver metabolome profiles of the double-crested cormorant (*Phalacrocorax auritus*), a large (1.5 to 3.0 kg) diving waterbird common in the Gulf of Mexico. Metabolomics analysis of avian plasma showed significant negative effects on avian metabolic profiles, in some cases after only two external exposures (26 g cumulative) to oil. We observed significant (p < 0.05) changes in intermediate metabolites of energy metabolism and fatty acid and amino acid metabolic pathways in cormorants after repeated exposure to oil. Exposure to oil increased several metabolites (glycine, betaine, serine and methionine) that are essential to the one-carbon metabolism pathway. Lipid metabolism was affected, causing an increase in production of ketone bodies, suggesting lipids were used as an alternative energy source for energy production in oil exposed birds. In addition, metabolites associated with hepatic bile acid metabolism were affected by oil exposure which was correlated with changes observed in bile acids in exposed birds. These changes at the most basic level of phenotypic expression caused by sublethal exposure to oil can have effects that would be detrimental to reproduction, migration, and survival in avian species.

## Introduction

In the subtropical Gulf of Mexico deep-water oil drilling accounts for more than 80% of all new drilling^1^. Inherent in this development in oil drilling practices have been increased risk of spills and changes in how these spills affect the environment^1,2^. The Deepwater Horizon (DWH) oil spill in April 2010 brought forth many of these differences in deep-water drilling and its environmental effects. The DWH oil spill was the largest offshore marine oil spill in the world and unprecedented in terms of loss of human life and economic and environmental impacts^1,3^. Key among these differences is that the oil travelled hundreds of meters through the water column, from 66 km offshore, and the active spill continued for several months, ultimately covering 112,100 km^2^ and exposing 2,100 km of shoreline off the coasts of Louisiana, Mississippi, Alabama and western Florida, USA^1,3^.

Large numbers of mortalities of wildlife including avian species have been documented in oil spills^4–6^ and the DWH oil spill was no exception^3^. However, the DWH oil spill was unique for a number of reasons. The nature of the DWH spill resulted in potentially repeated sublethal exposure to oil for weeks or even months post-spill over a wide geographic area^2,3,7^. Sublethal exposure was corroborated by many bird species seen alive during the DWH Natural Resource Damage Assessment^3,8^ but observed with moderate to trace amounts of oiling (<40% body coverage^5^). In addition, oil from the DWH Mississippi Canyon 252 (MC252) spill has been suggested to be more biodegradable and less toxic due to its chemical makeup^9,10^. The DWH event also occurred in warm sub-tropical waters and these factors presented unique characteristics associated with sublethal exposure to oil. So while previous research has indicated that sublethal dosages of oil can cause a wide range of adverse effects^11–14^ further research was needed to better understand the effects of repeated sublethal exposure of DHW oil on avian health and potential fate.

Our research builds on a recent suite of studies examining effects of exposure to DWH MC252 oil on various analytes and their physiological endpoints in multiple bird species^3^. In these studies, exposure to weathered MC252 oil resulted in changes in hematologic parameters, effects on multiple organs, cardiac function, feather damage, and increased heat loss and energetic demands^15–18^. In the current study, we further examine samples collected from an external oil dosing study conducted on Double-crested Cormorants (*Phalacrocorax auritus*; cormorant^19,20^) to evaluate the effect of exposure to DWH MC252 oil on the avian metabolome. Metabolomics provides a comprehensive evaluation of low molecular weight metabolites in a cell or organism and can be useful for understanding how metabolism and metabolic pathways of a biological system are affected when disturbed^21,22^. Because metabolites are closely linked to the phenotype of an organism they can provide basic functional information on physiological state^22–24^.

We assessed adverse effects of external sublethal exposure of DWH MC252 oil on metabolites extracted from plasma and the liver of Double-crested Cormorants, a large (1.2–2.5 kg), fish-eating, diving waterbird and a Gulf of Mexico relevant avian species^25^. Specifically we evaluated 49 metabolites in plasma and liver tissues affecting energy, fatty acid, amino acid and nucleoside metabolism. Our objective was to determine if repeated external application of sublethal amounts of artificially weathered MC252 oil to cormorants resulted in measurable changes to the metabolome profile of cormorants.

## Results

Monocytosis (>2.0 × 10^9^ cells/l) was noted in blood samples collected during the quarantine period from seven cormorants^16^. These cormorants were distributed at random between the control group (n = 4) and treatment group (n = 3). During the course of the trial, one bird from the control group died on day 1 and two birds from the treatment group died on days 14 and 19 and were not replaced. Plasma analyses included all birds sampled at each indicated time point and 11 birds in each group sampled for liver metabolome analyses at the end of the study. Food consumption declined throughout the quarantine period for both treatment and control groups. Food consumption continued to decline for control cormorants throughout the trial phase but increased for oiled cormorants which consumed on average 8.5% per day (33.6 g/d) more than the control group. Individuals in both groups gained weight over the study. Previously published research provides further details on total food consumption, changes in body weight, other clinical signs, and mortality^19^.

### Plasma Metabolome Profiles

**Figure 1:**
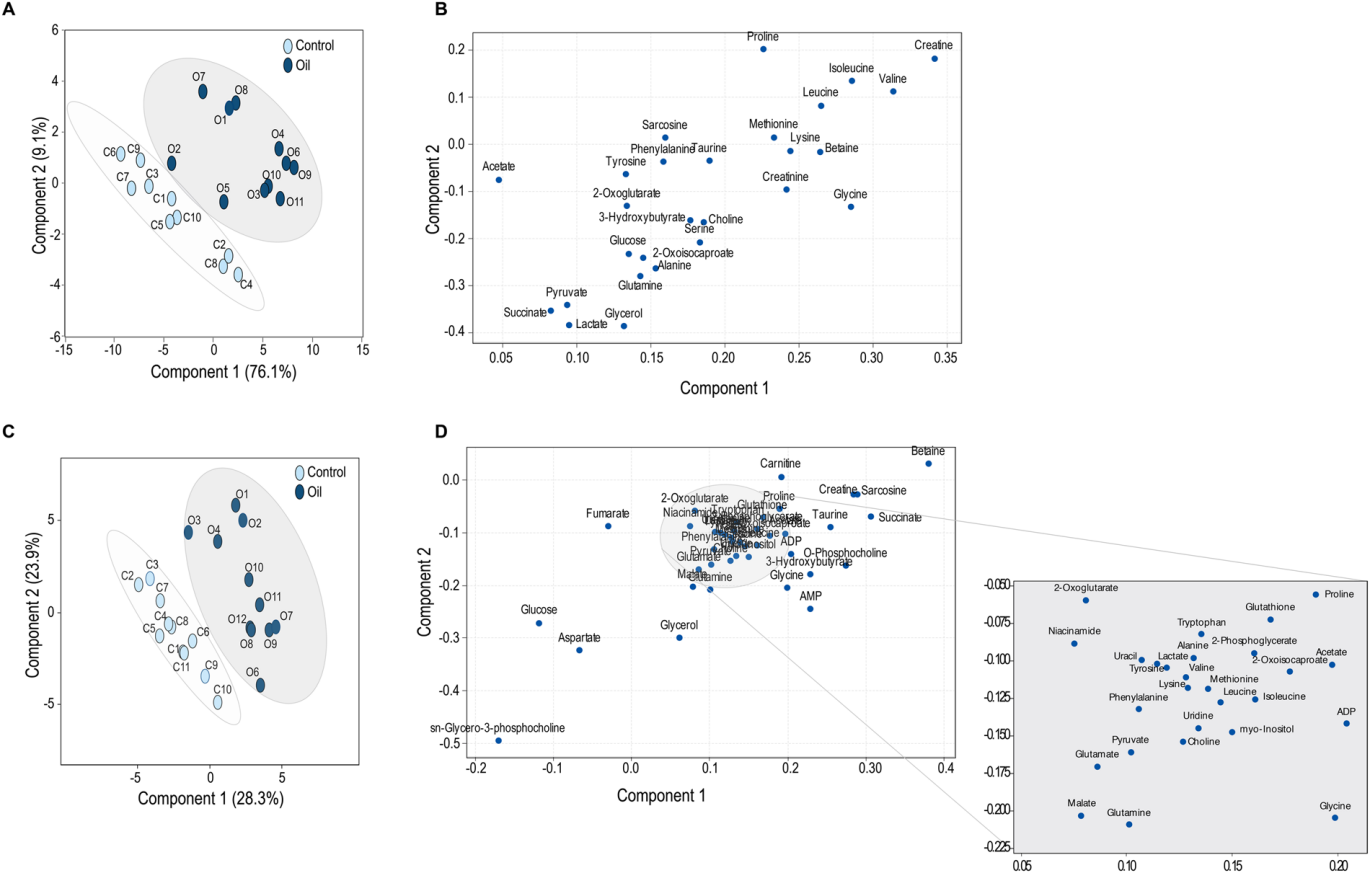
Multivariate PLSDA analysis of plasma and liver metabolites on the last day of repeated sublethal exposure of Deepwater Horizon, Mississippi Canyon 252 oil in Double-crested Cormorants (*Phalacrocorax auritus*). Panels A and C represent PLSDA score plots of overall differences in plasma and liver metabolome profiles, respectively between oiled and unoiled birds. Panels B and D provide PLSDA loading plots of the actual contributions of each metabolite to explaining between group variation in plasma and liver metabolome profiles (values closer to zero explain less variation).

The Nuclear Magnetic Resonance (NMR) plasma data revealed multiple changes in amino acid metabolism in oil-treated birds relative to control birds (Table 1). The partial least square discriminant analysis (PLSDA) clearly shows separation between two groups (oil-treated and control) for both tissues indicating a marked effect of oil treatment on metabolome phenotype (Fig. 1). Most of the metabolites with large differences between treated and control groups are common between both plasma and liver samples. These include branched chain, aromatic and essential amino acids in addition to metabolites belonging to tricarboxylic acid cycle (TCA) and fatty acid oxidation (Fig. 1).

**Table 1:** Plasma metabolites evaluated for effects of repeated exposure of Deepwater Horizon, Mississippi Canyon 252 oil on the plasma metabolome of Double-crested Cormorants (*Phalacrocorax auritus*).

Amino acid metabolism intermediates	Energy metabolism intermediates	Fatty acid metabolism intermediates
**PLASMA METABOLOME**		
2-Oxoisocaproate	2-Oxoglutarate	3-Hydroxybutyrate (3HB)^*^
Alanine^*^	Creatine^*^	Acetate^+^
Betaine^*,+^	Creatinine^*^	Choline^+^
Glutamine	Glucose	Formate
Glycine^*,+^	Lactate	Glycerol
Isoleucine^*^	Pyruvate	
Leucine^*^	Succinate	
Lysine		
Methionine^*,+^		
Phenylalanine^*^		
Proline^*^		
Sarcosine^+^		
Serine^+^		
Taurine^+^		
Tyrosine		
Valine		

*Indicates p < 0.05; ^+^indicates intermediates in one carbon cycle and histone/DNA modification.

**Figure 2:**
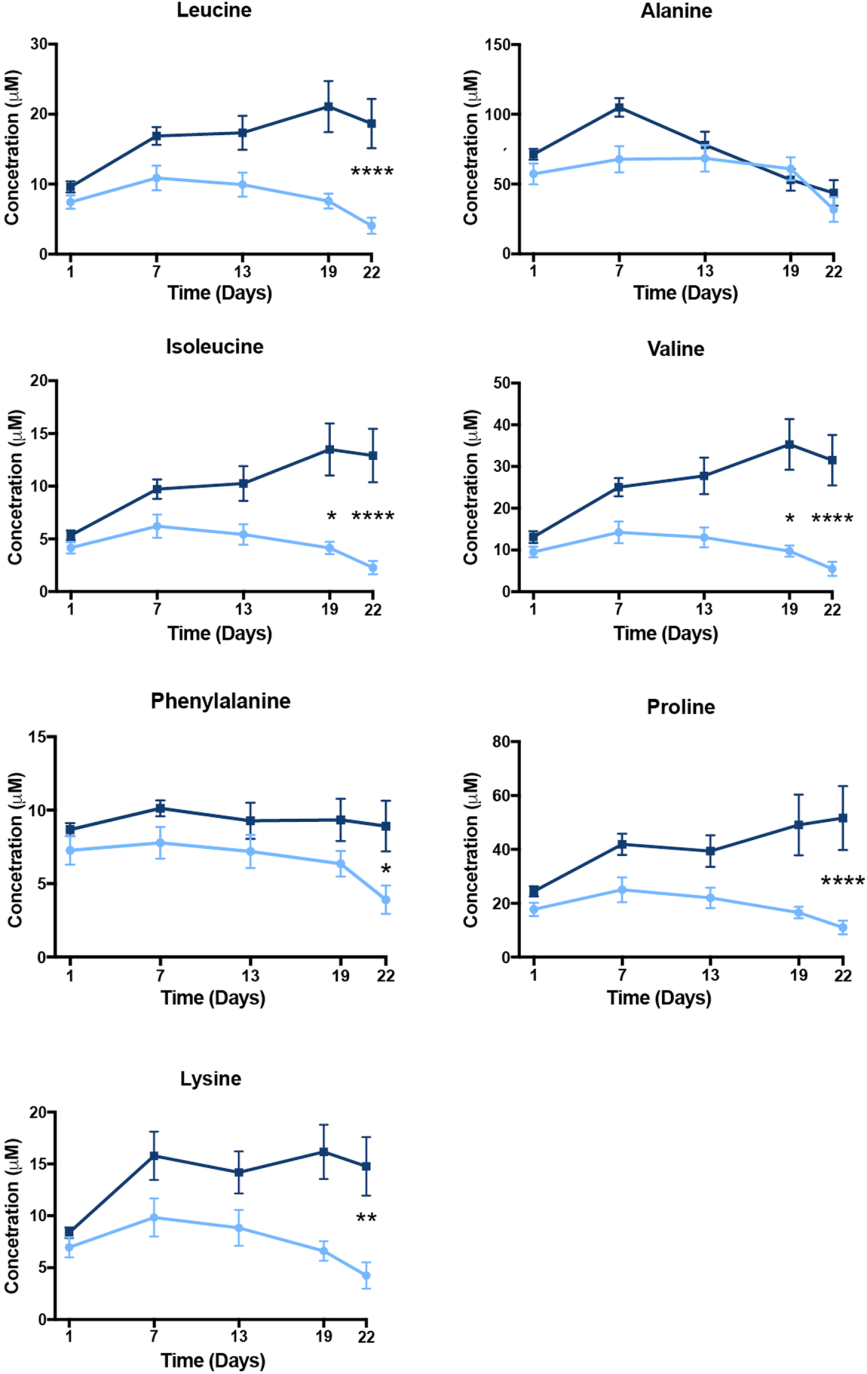
Plasma amino acid metabolites evaluated for effects of repeated sublethal exposure of Deepwater Horizon, Mississippi Canyon 252 oil to the plasma metabolome of Double-crested Cormorants (*Phalacrocorax auritus*). Data are shown as mean ± SEM, asterisks “*”, “**”, and “****” represent p < 0.05, p < 0.01, and p < 0.0001, respectively. Control are light blue and treated are dark blue lines.

**Figure 3:**
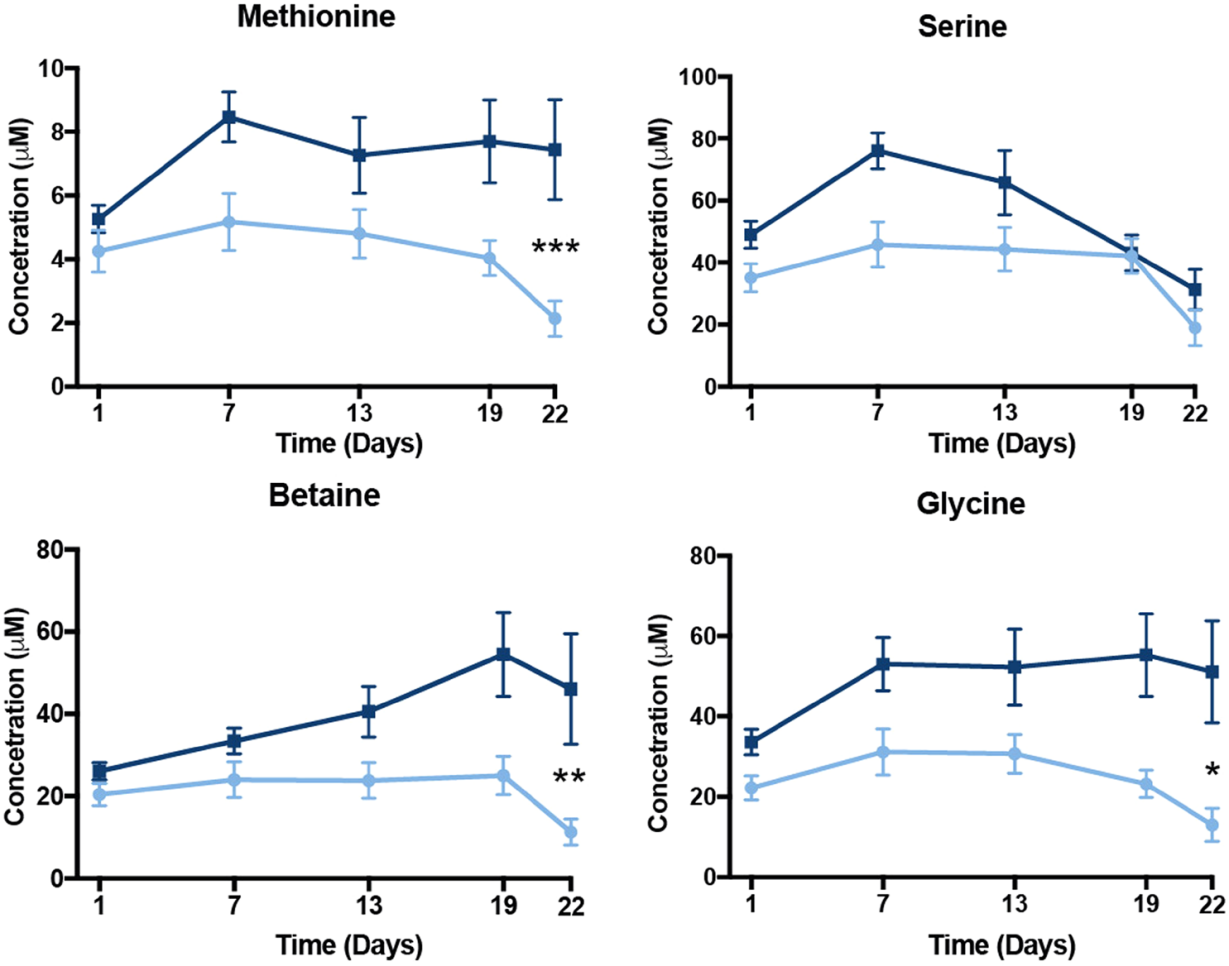
Plasma one-carbon metabolism pathway metabolites evaluated for effects of repeated sublethal exposure of Deepwater Horizon, Mississippi Canyon 252 oil to the plasma metabolome of Double-crested Cormorants (*Phalacrocorax auritus*). Data are shown as mean ± SEM, asterisks “*”, “**”, and “***” represent p < 0.05, p < 0.01, and p < 0.001, respectively. Control and treated are in light blue and dark blue lines, respectively.

In particular, levels of branched amino acids (isoleucine, leucine, valine) and other essential and non-essential amino acids (lysine, phenylalanine, proline) were increased as early as day 7 and continued to increase significantly (p < 0.05) by days 19 and 22 due to oil exposure (Fig. 2). In addition, the intermediate metabolites (glycine, betaine) essential to one-carbon cycle metabolism showed significant increased levels in the oil treated birds at 19 and 22 days (Fig. 3). The plasma level of methionine, an essential amino acid in the one-carbon cycle and for feather growth, was increased as early as the second oil exposure and reached significance (p < 0.05) by the last day of exposure (Fig. 3).

**Figure 4:**
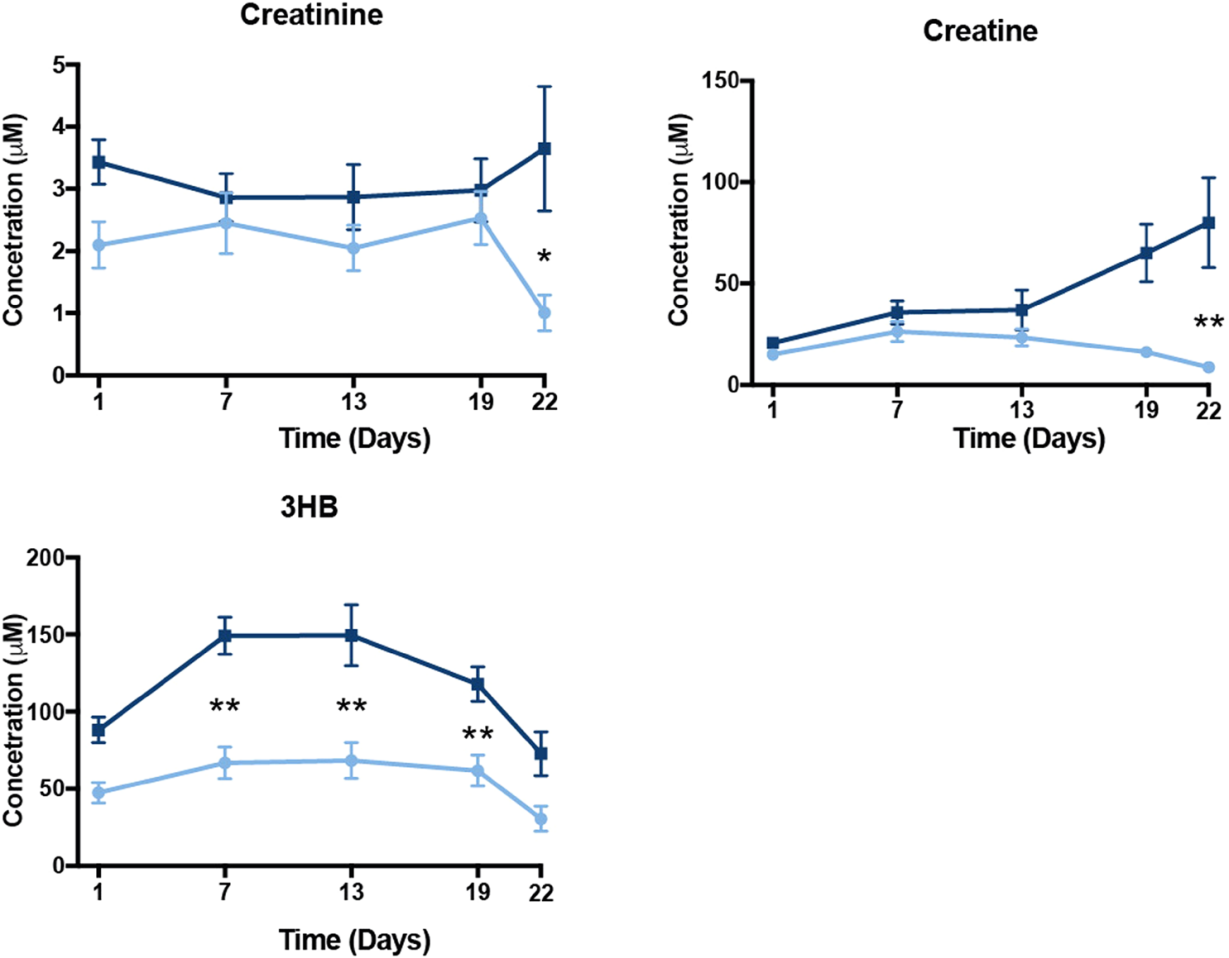
Carbon sources for energy metabolism evaluated for effects of repeated sublethal exposure of Deepwater Horizon, Mississippi Canyon 252 oil to the plasma metabolome of Double-crested Cormorants (*Phalacrocorax auritus*). Data are shown as mean ± SEM, asterisks “*”, and “**” represent p < 0.05 and p < 0.01, respectively. Controls are light in blue and oil-treated are in dark blue lines.

**Figure 5:**
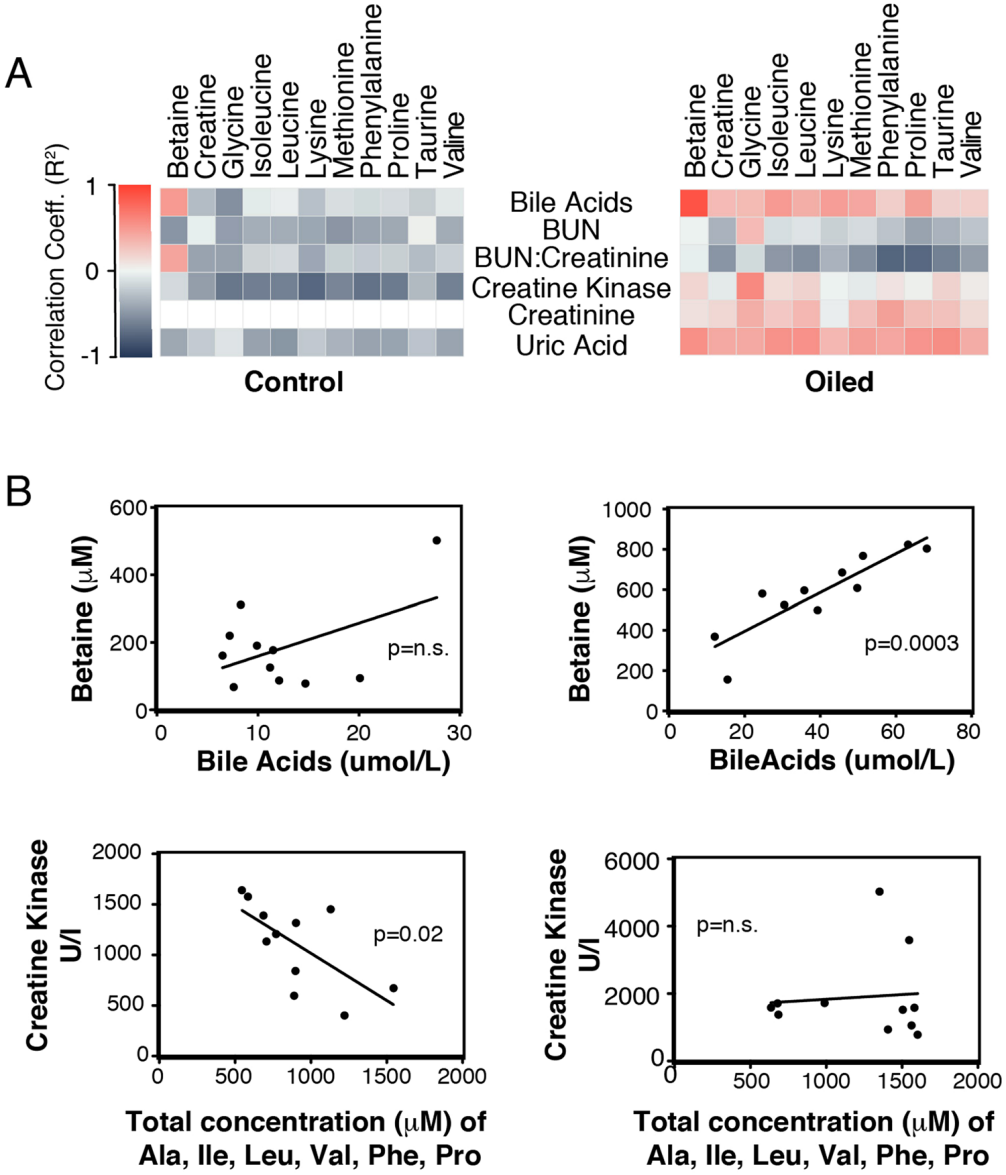
Pearson correlation plots for control and oil-treated animals. (**A**) Correlation heatmaps for control (left, n = 11 for each analyte) and oiled (right, n = 11 for each analyte) Double-crested Cormorants (*Phalacrocorax auritus*). Red shades indicate positive correlation, gray shades indicate negative correlation between each pair. X axis shows metabolites detected by 1D ^1^H NMR and Y axis shows enzymatic measurement^15^ in plasma samples on Days 22-23 of the experiment. (**B**) Correlation plots of selected pairs of measurements. P-values are indicated in the plots as the significance of the correlation between each correlation pair (n.s = p > 0.05).

We found increases in creatine and creatinine, biomarkers of kidney function and byproducts of amino acid degradation, in the plasma metabolome of oil-exposed birds (Fig. 4). Surprisingly, despite a non-fasting condition, plasma levels of 3-hydroxybutyrate (3HB), an end product of fatty acid oxidation, increased in treated birds as early as day 7 and remained significantly higher until day 22; the last day of the study (Fig. 4). This change indicates a significant shift in energy metabolism towards lipid oxidation despite high availability of carbon resources (i.e. amino acids and normal glucose level) in plasma and liver. Lastly, we found changes in correlations between oiled and unoiled birds for multiple affected intermediate metabolites (e.g. betaine) and physiological assay data (e.g. bile acids) from these same test subjects (Fig. 5A,B).

### Liver Metabolome Profiles

**Figure 6:**
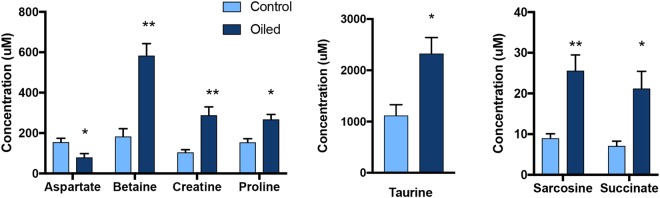
Liver amino acid metabolites evaluated for effects of repeated sublethal exposure of Deepwater Horizon, Mississippi Canyon 252 oil to the liver metabolome of Double-crested Cormorants (*Phalacrocorax auritus*). Data are shown as mean ± SEM, asterisks “*”, and “**” represent p < 0.05 and p < 0.01, respectively.

Several liver metabolites were significantly (p < 0.05) different between control and oil exposed birds, and in particular impairment of mitochondrial energy metabolism (Table 2). Levels of creatine and the TCA intermediate, succinate were significantly increased in the livers of oil-exposed birds (Fig. 6). Aspartate, which is synthesized from another TCA cycle intermediate oxaloacetate or alternatively used as a carbon source in TCA, was significantly reduced in the liver (Fig. 6). Consistent with plasma data, levels of betaine and taurine (intermediates in bile acid metabolism and/or contributing substrates to one-carbon cycle) were increased in the oil-treated birds (Fig. 6). Changes in creatine and sarcosine (intermediates in one-carbon metabolism) in the liver were also consistent with observed changes in the plasma metabolome (Figs 2–4).

**Table 2:** Liver metabolites evaluated for effects of repeated exposure of Deepwater Horizon, Mississippi Canyon 252 oil on the liver metabolome of Double-crested Cormorants (*Phalacrocorax auritus*).

Amino acid metabolism intermediates	Energy metabolism intermediates	Fatty acid metabolism intermediates	Nucleoside metabolism intermediates
**LIVER METABLOME**			
2-Oxoisocaproate	2-Oxoglutarate	3-Hydroxybutyrate	Uracil
Alanine	2-Phosphoglycerate	Acetate^+^	Uridine
Aspartate^*^	ADP	Carnitine	
Betaine^*,+^	AMP	Choline^+^	
Glutamate	Creatine^*^	Formate	
Glutamine	Fumarate	Glycerol	
Glutathione^+^	Glucose	O-Phosphocholine^+^	
Glycine^+^	Lactate	sn-Glycero-3-phosphocholine^+^	
Isoleucine	Malate		
Leucine	myo-Inositol^+^		
Lysine	Pyruvate		
Methionine^+^	Succinate^*^		
Niacinamide (NAD)^+^			
Phenylalanine			
Proline^*^			
Sarcosine^*,+^			
Taurine^*,+^			
Tryptophan^+^			
Tyrosine			
Valine			

*Indicates p < 0.05; ^+^indicates intermediates in one carbon cycle and histone/DNA modification.

## Discussion

Evaluation of the effects of repeated sublethal exposure to oil on the cormorant’s metabolome provides new molecular insights into our previous findings of physiological responses to oil exposure. Despite characteristics of the DWH oil being described as potentially less toxic and more biodegradable than other oil sources^9,10^, our current metabolomics study indicated that repeated sublethal exposure to this oil negatively impacts avian physiology at basic levels of biochemical phenotypic expression. We observed changes in multiple metabolic pathways associated with impairment in energy production from fatty acids and amino acids metabolism in both plasma and liver samples in oil exposed birds (Fig. 1). With respect to plasma samples, only 3HB was significantly affected after as little as two applications of oil (26 g total) applied prior to blood sampling on day 6 (Fig. 4), while others such as branched and aromatic amino acids started to show an early increase followed by statistically significant impacts after several exposures (days 19 to 22, Figs 2 and 3).

As a carnivore, piscivorous birds obtain most of their energy from a protein rich diet. Amino acids, the building blocks of proteins, play a major role in maintaining blood glucose concentrations, energy generation and serve as structural elements for insulation such as feathers. The increased blood levels of branched amino acids, leucine, isoleucine and valine in circulation have been previously linked to impaired insulin regulation and increased risk of insulin resistance and type-2 diabetics in rodents and humans^26^. Alternatively, amino acids could be utilized as a carbon source for energy metabolism to produce heat from ATP. Isoleucine and valine are catabolized into intermediates that could be readily used in the tricarboxylic acid (TCA) cycle to meet energy requirements.

Contrary to our expectation, oil treatment had a significant impact on fatty acid utilization in these birds. Our data shows that oil exposed birds maintained blood glucose at the expense of alterations in amino acid utilization and increased fatty acid oxidation under non-fasting condition. The plasma biochemical metabolome showed that repeated oil exposure increased several key amino acid metabolites (glycine, betaine, serine, and methionine) essential in one-carbon metabolism (Fig. 3). One-carbon metabolism is a complex multi-compartmental pathway that is the major supplier of methyl groups for biological reactions^27^. For example, the metabolite betaine was affected by oil exposure in birds as indicated by 1D ^1^H NMR and this disruption was significantly correlated with changes in bile acids in oiled birds (Fig. 5A,B). These bile acids are in the liver, and together with betaine are involved in one-carbon cycle pathway. Alterations in this pathway have an impact on epigenetic mechanisms that can affect gene expression through DNA methylation and therefore cellular function of many proteins^28^.

**Figure 7:**
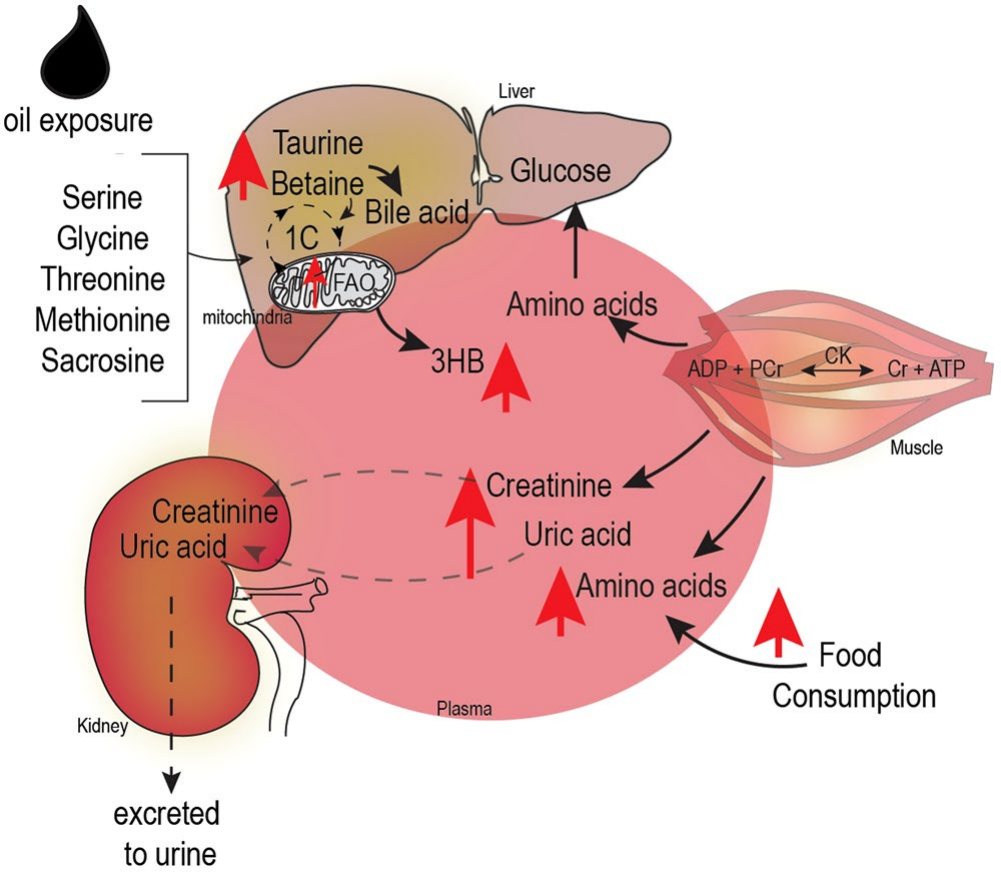
Summary of metabolic changes in Double-crested Cormorants (*Phalacrocorax auritus*) due to repeated sublethal external exposure to Deep Water Horizon, Mississippi Canyon 252 oil indicate increased amino acid metabolism and lipid oxidation in tissues (i.e. plasma, liver, muscle and kidneys). Net increased amino acid levels in plasma (i.e. BCAA and aromatic amino acids) was in part, contributed by protein breakdown in muscles as was supported by increased creatinine kinase activity (CK) in blood^15^. Oil exposure also increased levels of creatinine (Cr) and uric acid (end products of nitrogen metabolism) supporting consequent impairment in kidney function due to increased amino acid utilization. In the liver, serine, methionine, glycine, and sarcosine, part of the one carbon cycle (1C) and bile acid production pathways, increased. These amino acids are also coupled to an increased demand for fatty acid oxidation (FAO) that led to increased levels of ketone body, 3-hydroxybuyrate (3HB) in plasma. Only those metabolites with statistically significant change (p < 0.05) are shown, (PCr = phosphocreatine).

Figure 7 shows a summary of effects of changes in the untargeted metabolome profiles of oil-treated birds. Three key metabolic changes are observed in plasma and liver that include increased fatty acid oxidation and amino acid metabolism. The increased amino acids and creatinine could partly result from protein breakdown in muscles as supported by previous research that showed increased creatinine kinase activity and uric acid in blood of oil treated birds^15^ although we could not exclude contribution from increased food consumption.

Alterations of creatine metabolism and its intermediates could provide information on the muscle energy balance and pathological changes in renal function^29^. Creatine and creatinine, byproducts of amino acid metabolism, have established roles in energy metabolism, supplying energy in the form of ATP^29^. Creatine could be utilized during nutrient stress and turbulent energy demand caused by oil exposure to sustain oxidative phosphorylation^30^ by creatine kinase (CK). Creatine kinase is an enzyme that through a reversible reaction could generate creatine (Cr) from phospho-creatine (PCr) to produce ATP (Fig. 7). Previous biochemical assays^15^ showed increased CK levels and uric acid excretion (the end product of creatinine metabolism). Our correlation analysis between CK and amino acid levels showed differences in animals exposed to sublethal dosages of oil (Fig. 5A,B). In blood, increased CK level is a marker of muscle damage. The strong negative correlation between plasma CK levels and amino acids including branch amino acids was lost due to the oil exposure. This result supports our theory that an altered nitrogen balance resulted in increased amino acid utilization in oil exposed birds with the same diet regimes as unoiled birds in order to maintain the increased demand for energy metabolism (Fig. 7).

Another surprising finding in the plasma metabolome was the increased level of ketone body, 3HB, in oil-exposed birds under ad libitum feeding. Plasma 3-hydroxybutyrate is a well-established indicator of nutrient stress that induces glucose sparing for the brain, while allowing peripheral organs to use 3HB for energy generation^31^. In addition, the elevated level of 3HB in plasma is associated with hepatic mitochondrial metabolism^32^ and a key signature of mitochondrial impaired rates of hepatic ATP synthesis^33^ and insulin sensing^34^ under nutrient stress response.

Further support of energy distress comes from damaged feathers in oil treated birds that showed an increased metabolic rate due to insufficient insulation^35^. Cormorants exposed to oil in this study exhibited increased heat loss, difficulty in maintaining thermoregulatory balance, hyperplastic goiter of the thyroid, and increased food consumption^17–19^. Observational data shows increased feather plucking and degradation in oil-exposed birds^19^. This resulted in loss of feather insulation and increased heat loss in exposed birds compared to control animals^18^. The presence of high levels of methionine in plasma may be a physiological response to observed feather degradation and feather plucking, since methionine is a sulfur containing precursor of cysteine; one of the highly abundant amino acids in feather keratins^36^. Based on our metabolome data, we postulate that oil exposure induced a nutrient and/or energy stress response to induce fatty acid oxidation possibly through 3HB signaling to compensate the energy demands of these animals, even though they are in a fed state.

Energy metabolism is also closely linked to thermogenesis, therefore, the increased energy demand and the shift in the substrate utilization could be partially explained by the increased need for maintaining the body temperature in those oil exposed birds. Similar exposure to DWH oil resulted in lower body mass and alterations to flight paths in Rock Pigeons (*Columba livia*)^37,38^ and increased energetic demands associated with flight take-off in Western Sandpipers (*Calidris mauri*)^39^. This study and others provide evidence that sublethal exposure to DWH oil not only resulted in physical damage to feathers but can cause reduced efficiency in energy production due to disruption of energetic pathways and can have profound effects on behavior and physiological function of avian species. For example, migrating birds rely on lipid metabolism and fatty acid oxidation to provide fuel to muscles during extended flights^40^. Disruption of lipid oxidation pathways, which we observed in resting birds exposed to oil, could prevent bioenergetic demands required for migration to be met, and lengthen time required to complete migration and delay migration arrival dates.

Liver metabolomics data in this study was consistent with what was observed in the plasma metabolome and indicated that hepatic bile acid metabolism in mitochondria was significantly disturbed by oil exposure. Previous research has documented abnormal, pale yellow bile along with increased mass of liver and kidney, as well as liver damage^17^. Furthermore, gastrointestinal edema were observed at necropsy in cormorants orally dosed with DWH oil^17^. In this study, we detected increased levels of betaine and taurine (Fig. 4), which supports that oil exposure altered the hepatic bile acid metabolism. Though the current data could not fully explain the relationship between altered liver structure and bile acid metabolism in oil exposed birds, we can postulate that in part the increased betaine may feedback to the one carbon cycle that is tightly coupled to histone acetylation, and thus induce epigenetic consequences.

Factors such as increased levels of acetate and ketone bodies such as 3-hydroxybutyrate are shown to be signaling molecules that influence histone modification^41^. Increased 3HB is shown to inhibit the closed form of chromatin and allow increased histone acetylation leading to increased lipid oxidation consistent with observed increased levels of 3HB^41^. Our metabolome data indicated that the level of 3HB in plasma significantly increased after the second treatment with DWH oil and remained high until the last day of exposure. Future studies are needed to evaluate the role of 3HB and one carbon cycle in epigenetic remodeling of histone proteins and DNA modifications due to oil exposure.

In summary, exposure to sublethal levels of oil altered and disrupted multiple metabolic pathways in both the plasma and liver metabolome of cormorants. These findings are consistent with the existing literature indicating negative impacts to thermoregulation, multiple organ systems, cardiac function, and hematologic parameters in Double-crested Cormorants^3,15–17^. Given this result, we recognize that we investigated a single repeated dosing rate of oil over a three-week period, which represents only a few points on a continuum of possible oil exposure scenarios. Untargeted metabolomics in additional oil damaged tissues (kidney, heart, bile, and gut tissue) and targeted, functional assay NMR-metabolomics could provide information on how flux in energy metabolism is changed and metabolic pathways are altered or disrupted through oil exposure. Such research done across a range of dose responses could provide useful mechanistic information in determining impacts to wildlife species affected by oil spills.

Sublethal external exposure to petroleum crude oil from spills can have significant detrimental impacts to the avian metabolome. Several metabolic pathways are affected which may result in impairment of energy, fatty acid, and amino acid metabolism in both plasma and liver, and likely other organs as well. These changes at the most basic level of phenotypic expression can have effects that would be detrimental to migration, reproduction and survival in avian species. Given our findings and related research, field assessments of damage due to oil spills likely underestimate the true cost in terms of both individual and population level effects on avian species.

## Methods

### Animal Husbandry

This work is a continuation of a series of live bird experiments so we include these methods to provide necessary context. Additional detail can be found in previous research^19^. A total of 31 cormorants were captured from boats at night^42^ from the wild in December 2014 and January 2015 from Alabama (AL) and Mississippi (MS). All animals were captured under Federal Collection Permit MB019065, and MS and AL (#8017) scientific collection permits and housed at the U.S. Department of Agriculture, Wildlife Services, National Wildlife Research Center (NWRC) facilities in Starkville, MS. Cormorants were individually housed in 3.3 m × 1.5 m × 2.0 m (length x width x height) cages, each of which was provided with a 190-liter plastic tank filled with water. The water was changed daily to limit oil re-exposure. Live channel catfish (*Ictalurus punctatus*) were provided as a food source (600 g/day). All uneaten catfish were removed from individual tanks daily and weighed to assess individual daily food consumption. Cormorants were acclimated to captivity for at least 21days prior to initiation of the study.

### Ethic statement

This research was conducted in accordance with the USDA guidelines of animal care and use. All experimental protocols of animal capture and handling were approved by the United States Department of Agriculture (USDA), National Wildlife Research Center, Institutional Animal Care and Use Committee (IACUC) Protocol QA-2326 and NWRC Attending Veterinarian review.

### Treatments and Dosing

The oil used was artificially weathered Mississippi Canyon 252 (MC252) oil (DWH7937, batch# B030112) prepared from crude oil collected during the DWH oil spill. The oil was artificially weathered to provide a chemical profile similar to oil birds would be exposed to from the DWH spill^10^. A total of 25 cormorants were allocated to two treatment groups; a control group (n = 12, 5 male, 7 female) and treatment group (n = 13, 6 males, 7 females). The treatment group was externally oiled over approximately 20% of their surface area, exclusive of tail feathers and wings^19^. This oiling rate was the upper limit of the light oiling category (6–20% coverage) for live birds observed in the Natural Resource Damage Assessment by the U.S. Fish and Wildlife Service for the DWH oil spill^3^. To achieve this coverage, oil was applied to the breast and back feathers using plastic stencils, which measured 8 × 17.5 cm and 7 × 20 cm, respectively. The weight of oil applied to each bird during each application totaled 13 g, with approximately 6.5 g applied to the breast and 6.5 g applied to the back. The control group received 6.5 g of water applied to the breast and 6.5 g of water applied to the back to ensure similar treatment and handling as oiled cormorants. Treatments were applied every three days through Day 15 of the trial (on days 0, 3, 6, 8, 12, and 15)^19^.

All cormorants had a blood sample taken via jugular, brachial, or tarsal venipuncture 21 days prior to study initiation to provide baseline data. Complete blood count (CBC) values were used to ensure equal division of cormorants with potential preexisting conditions^16^. Body weight and condition were monitored every three days when birds were handled. Observational health checks were conducted twice daily. Once testing began, blood was collected every six days (days 0, 6, 12 and 18), prior to external application of oil. Blood for plasma was collected using syringes flushed with 100 U/ml lithium heparin and placed into vacutainer tubes with lithium heparin additive.

### Necropsy

At the end of the trial, cormorants were sampled for blood (as above), euthanized, and necropsied on days 21 and 22 of the study. Cormorants were euthanized using cervical dislocation and necropsied immediately thereafter. The liver and other organs were collected from all cormorants and sex was identified and recorded^17^. Liver samples (~0.5 g) from each cormorant were weighed to the nearest 0.1 mg, placed in labeled cryovials, flash frozen with liquid nitrogen.

### Metabolomics

Plasma metabolites were extracted by adding 2 volumes of methanol into 50 µl of plasma samples, vortexed and kept at −20 °C for 30 min for protein precipitation according to our previously published protocol^20^. Samples were then centrifuged at 5,000 g for 15 minutes at 4 °C. The supernatant was dried overnight by speed-vac. The dried supernatant was then reconstituted in the tissue NMR buffer (i.e., 20 mM phosphate in D2O (99.9% ^2^H), containing 1 mM formate, 0.5 mM 4,4-dimethyl-4-silapentane-1-sulfonic acid (DSS), and 0.1 mM NaF) and pH was adjusted to 7.4 ± 0.05.

Samples of 50 mg of liver tissue were homogenized in 20 mM phosphate buffer using Omni Homogenizer (Omni International Inc. Waterbury, Conn.)^43^. Homogenates were centrifuged at 5,000 g, for 10 minutes at 4 °C. Supernatants were transferred to a 2 ml Eppendorf tube and proteins were precipitated with two volumes of methanol, vortexed and kept at −20 °C for 30 min. Cold samples were centrifuged at 5,000 g, for 15 minutes at 4 °C. Supernatants were transferred to a new 2 ml tube and dried by speed vaccum overnight. The dried supernatant was reconstituted in the tissue NMR buffer (i.e. 99.9% ^2^H_2_O containing 1 mM formate, 0.5 mM DSS, and 0.1 mM NaF) and pH was adjusted to 7.4 ± 0.05.

### NMR data collection and processing

One dimensional proton nuclear magnetic resonance spectroscopy (1D ^1^H NMR) spectra of plasma and liver samples were collected at the NMR Facility at University of Wisconsin-Madison^21^. The 1D ^1^H NMR spectra were referenced to DSS as the internal chemical shift reference. Metabolite concentrations were quantified relative to 1 mM formate as internal reference using Chenomx software version 6 (http://www.chenomx.com) at the University of Wisconsin- Madison. The analysis of NMR data identified and quantified twenty-eight metabolites in plasma (Table 1) and forty-two metabolites in liver (Table 2) as a result of repeated sublethal exposure of DWH oil to cormorants. Prior to visualization, NMR output data was subjected to pre-treatment by centering and scaling, and followed by log_2_‐transformation^44^.

### Statistical analysis

Changes in the plasma and liver metabolome of the oil treatment and control groups (n = 11 birds/group) were evaluated using a partial least square discriminant analysis (PLSDA)^45–47^, to identify a subset of metabolites as key players contributing to variance between groups. We used PLSDA due to the following reasons: 1) it is highly suited for the analysis of high dimensional data sets characterized by low sample size and many variables, 2) PLSDA allows the capture of information regarding how each predictive variable (in these terms metabolites) is related to the dependent variable (oil treatment or control) with reduced dimensions (number of metabolites) providing stronger analysis^45–47^. We applied PLSDA to metabolome profiles on the last day of oil treatment (day 22 or day 23) and compared control and treatment groups for both plasma and liver. We reported two components of PLSDA; score plots and loading plots (see Supplementary Methods for further PLSDA model explanation and validation information).

To better understand magnitude and direction of changes we also analyzed plasma data by repeated measures ANOVA and liver data by t-test. Plasma metabolome profiles of oiled (n = 13) and control animals (n = 12) were also evaluated by repeated measures ANOVA and significances were assigned by Tukey’s multiple comparisons test. Plasma ANOVA models included main effects for treatment, elapsed days, and their interaction (treatment x day). Liver data was analyzed by Student’s t-test and false discovery rate^48,49^ to evaluate differences in metabolome profiles of oiled (n = 11) and control birds (n = 11). Data are presented as mean ± SEM for relative concentrations of metabolites to the internal standard (formate). Lastly, to investigate support of disruption of specific metabolic pathways, we investigated the correlation between plasma metabolites identified as disrupted by oil exposure by 1D ^1^H NMR and previously reported plasma assays^15^ using a Pearson correlation analysis using R (R Core Team 2013)^50^.

## Electronic supplementary material

Supplementary Information

## Data Availability

Data for captive animal and sample collection portion of the study conducted as part of the Deepwater Horizon Damage Assessment are publicly available at https://www.diver.orr.noaa.gov/deepwater-horizon-nrda-data. Data from NMR analyses are archived with U.S. Department of Agriculture, Wildlife Services, National Wildlife Research Center under QA-2326 and available from the corresponding author on reasonable request.
