# Supplementary material for: Effects of Repeated Sublethal External Exposure to Deep Water Horizon Oil on the Avian Metabolome
Source: Sci Rep. 2019 Jan 23;9:371. doi: 10.1038/s41598-018-36688-3 (PMC6344488; doi:10.1038/s41598-018-36688-3)
Supplement: Supplementary file 1 — Supplementary Information [file 41598_2018_36688_MOESM1_ESM.pdf]

Supplementary File

Title: Effects of Repeated Sublethal External Exposure to Deep Water Horizon Oil on the Avian Metabolome

Brian S. Dorr[1a], Katie C. Hanson-Dorr[1], Fariba M. Assadi-Porter[2], Ebru Selin Selen[2], Katherine A. Healy[3], Katherine E. Horak[4]

[1]US Department of Agriculture, Wildlife Services, National Wildlife Research Center, MS State, MS 39762 USA

[2]Department of Integrative Biology, University of Wisconsin-Madison, Madison, WI 53706 USA

[3]US Fish and Wildlife Service, Deepwater Horizon Natural Resource Damage Assessment and Restoration Office, Fairhope, AL, 36532 USA

[4]US Department of Agriculture, Wildlife Services, National Wildlife Research Center 4101 LaPorte Ave, Fort Collins, CO 80521 USA

[a] Corresponding Author: Brian.s.dorr@aphis.usda.gov

Supplementary Methods

Changes in the plasma and tissue metabolome of the oil treatment and control groups were evaluated using a partial least square discriminant analysis (PLSDA) approach, to identify a subset of metabolites as key players contributing to variance between groups[1,2,3]. The matrix that is subjected to PLSDA consists of a descriptor matrix, where rows are carrying the samples, and variables (metabolites that include the concentration levels directly quantified from 1H-NMR experiment. The Y matrix on the other hand is a matrix of dummy variables describing the class membership (corresponding to experimental groups) of each observation (observation: experimental samples) in the descriptor matrix X. PLSDA is a form of partial least square regression that calculates latent variables as a linear combination of X in such way that they will approximate X and Y and maximize the covariance between X and Y.

In this study, we report two components of PLSDA; scores plots and loadings plots. Those plots are visual aids to examine the similarities and differences in a data set. In this case, we are examining a metabolomics data set. Therefore they provide a condensed summary of the original dataset. A scores plot shows a summary of the relationships among the observations, and the loadings plot shows a similar summary between the variables. A higher loadings value of a variable (i.e. a metabolite) corresponds to higher importance of the contribution of the metabolite to the variation between two groups[4]. A loadings plot is a medium to interpret the patterns seen in a score plot. The two plots are complementary and superimposable so the one direction in one plot will correspond to the same direction in the other plot. Those subset of metabolites with priority score with loading value > |0.19| are defined as the most contributing metabolites. The threshold is chosen according to the dataset and the loadings values. This subset was referred as the ‘reduced subset’ in later sections.

The other important aspect of the PLSDA approach is model validation. Model validation consists of two components; the first one is a parameter, Q2, for measuring the predictability of the model (separation observed in scores plot) and the other parameter R2 measures the quality of the model. The range of values that those parameters can be within the range of [0,1]. Values closer to 1 indicate good model fit between the dataset and the regression model[2,5,6,7,8].

Table 1. PLSDA Plasma Cross Validation

**Measure**	**1 comps**	**2 comps**	**3 comps**	**4 comps**	**5 comps**
Accuracy	0.7619	0.90476	0.90476	0.90476	0.90476
R2	0.47717	0.81449	0.88941	0.9254	0.94717
Q2	0.38474	0.7252	0.73592	0.71076	0.63774

Table 2. PLSDA Liver Cross Validation

**Measure**	**1 comps**	**2 comps**	**3 comps**	**4 comps**	**5 comps**
Accuracy	0.90909	1.0	0.95455	1.0	1.0
R2	0.86307	0.93719	0.97951	0.9869	0.99414
Q2	0.72363	0.78381	0.76333	0.79015	0.792

References

1. Lindgren, F., et al., *Model validation by permutation tests: Applications to variable selection.* Journal of Chemometrics, 1996. **10**(5-6): p. 521-532.
2. Teng, Q., *NMR-Based Metabolomics*, in *Structural Biology*2013, Springer US. p. 311-392.
3. Pattini, L., et al., *An integrated strategy in two-dimensional electrophoresis analysis able to identify discriminants between different clinical conditions.* Exp Biol Med (Maywood), 2008. **233**(4): p. 483-91.
4. Eriksson, L., et al., *Megavariate analysis of environmental QSAR data. Part II--investigating very complex problem formulations using hierarchical, non-linear and batch-wise extensions of PCA and PLS.* Mol Divers, 2006. **10**(2): p. 187-205.
5. Wold, S., M. Sjöström, and L. Eriksson, *PLS-regression: a basic tool of chemometrics.* Chemometrics and Intelligent Laboratory Systems, 2001. **58**(2): p. 109-130.
6. Li, M., et al., *Symbiotic gut microbes modulate human metabolic phenotypes.* Proc Natl Acad Sci U S A, 2008. **105**(6): p. 2117-22.

7 Barker, M. and W. Rayens, *Partial least squares for discrimination.* Journal of Chemometrics, 2003. **17**(3): p. 166-173.

8. Eriksson, L., *Multi-and megavariate data analysis*2006: MKS Umetrics AB.
